# Probing the Energy Landscape of Activation Gating of the Bacterial Potassium Channel KcsA

**DOI:** 10.1371/journal.pcbi.1003058

**Published:** 2013-05-02

**Authors:** Tobias Linder, Bert L. de Groot, Anna Stary-Weinzinger

**Affiliations:** 1 Department of Pharmacology and Toxicology, University of Vienna, Vienna, Austria; 2 Computational Biomolecular Dynamics Group, Max Planck Institute for Biophysical Chemistry, Göttingen, Germany; University of Illinois, United States of America

## Abstract

The bacterial potassium channel KcsA, which has been crystallized in several conformations, offers an ideal model to investigate activation gating of ion channels. In this study, essential dynamics simulations are applied to obtain insights into the transition pathways and the energy profile of KcsA pore gating. In agreement with previous hypotheses, our simulations reveal a two phasic activation gating process. In the first phase, local structural rearrangements in TM2 are observed leading to an intermediate channel conformation, followed by large structural rearrangements leading to full opening of KcsA. Conformational changes of a highly conserved phenylalanine, F114, at the bundle crossing region are crucial for the transition from a closed to an intermediate state. 3.9 µs umbrella sampling calculations reveal that there are two well-defined energy barriers dividing closed, intermediate, and open channel states. In agreement with mutational studies, the closed state was found to be energetically more favorable compared to the open state. Further, the simulations provide new insights into the dynamical coupling effects of F103 between the activation gate and the selectivity filter. Investigations on individual subunits support cooperativity of subunits during activation gating.

## Author Summary

Voltage gated ion channels are membrane embedded proteins that initiate electrical signaling upon changes in membrane potential. These channels are involved in biological key processes such as generation and propagation of nerve impulses. Mutations may lead to serious diseases such as cardiac arrhythmia, diabetes or migraines, rendering them important drug targets. The activity of ion channels is controlled by dynamic conformational changes that regulate ion flow through a central pore. This process, which involves opening and closing of the channels, is known as gating. To fully understand or to control ion channel gating, we need to unravel the underlying principles. Crystal structures, especially of K^+^ channels, have provided excellent insights into the conformation of different channel states. However, the transition states and structural rearrangements are still unknown. Here we use molecular dynamics simulations to simulate the full transition pathway and energy landscape of gating. Our results suggest that channel gating involves local structural changes followed by global conformational changes. The importance of many of the residues identified in our simulations is supported by experimental studies. The ability to accurately simulate the gating transitions of ion channels may be beneficial for a better understanding of ion channel related diseases and drug development.

## Introduction

**Figure 1:**
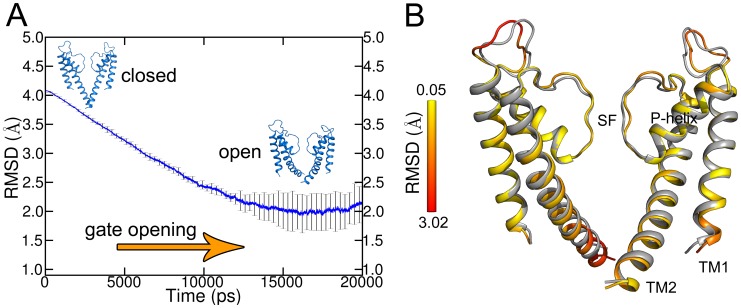
RMSD analysis of ED opening simulations. A) Average of the backbone RMSD (without loops) of ten opening ED simulations. The open crystal structure was used as reference. The standard deviation is indicated by error bars. B) Comparison of the average structure (built out of the minimal RMSD structures of the ten ED simulations; yellow to red) and the open crystal structure (gray). The RMSD of the Cα atoms is shown as a spectrum from yellow to red. For the sake of clarity, only the two opposite SUs are shown.

K^+^ channels play a crucial role in a wide variety of physiological and pathophysiological processes including action potential modeling [1], cancer cell proliferation [2], and metabolic pathways mediation [3]. In the last few decades, the understanding of ion channels has increased tremendously. The Hodgkin-Huxley equations [4] provided first insights into the ion flow in nerve cells and Hille showed a comprehensive picture of the electrophysiological properties of ion channels [5]. In 1998, the first crystal structure of an ion channel, the bacterial potassium channel of *Streptomyces lividans* (KcsA), shed light on the molecular details of a K^+^ channel [6]. The pore-forming domain of KcsA is composed of four identical subunits (SUs) which are arranged symmetrically around a channel pore. Each SU consists of two transmembrane helices, TM1 and TM2, which are connected by the P-helix and the selectivity filter (SF) (Figure 1B). While the extracellular facing SF tunes the selection of different ions and modulates inactivation, the main conformational changes regulating ion flow, are found at the TM2 helices. These motions, referred to as activation gating, are thought to involve an iris-like motion of the TM2 helices that constrict the permeation pathway at the helix bundle crossing region [7]–[10]. This region is believed to form the main activation gate. Starting in 1998, several different pore domain structures of KcsA in its closed state [6], [11] and more recently in intermediate and open states have been solved [12]. These crystal structures provide excellent insights into different conformations of proteins; however, they feature only snapshots of dynamical proteins [13]. Therefore, the transition steps and the mechanisms of activation gating are still unknown.

A number of computational studies have been published over the last years, aiming at exploring the gating pathways of ion channels by making use of available X-ray structures as templates [14]–[22]. However, the lack of particular K^+^ channels in different conformations was a limitation of previous publications. Thus, these studies had to compare crystal structures of different channels or had to rely on homology models of open structures of KcsA. With the successful crystallization of intermediate and open structures of KcsA by Cuello et al in 2010 [12], *in silico* activation gating of K^+^ channels cannot only be readdressed, but also allowed us to calculate a complete energy profile of activation gating. The essential dynamics (ED) simulation method has been shown as a useful tool to investigate sampling of proteins in conformational space and to derive transition pathways between conformational states [23]–[27]. In this study, we applied ED simulations combined with umbrella sampling calculations to investigate activation gating of KcsA.

## Results/Discussion

### Stability of closed and open conformations

A prerequisite of the ED method is that the starting and target structures are of equal length and identical amino acid sequence. Thus, the KcsA crystal structures (pdb identifier: 1k4c, closed; 3fb6, intermediate; 3f7v, open) were adjusted at the N- and C-termini so that all states started from residue 29 and ended at residue 118, leading to channels with four times 89 amino acids. Additionally, Q117 in the open and intermediate crystal structure was mutated to arginine to obtain the wild type structure.

Before probing the transition pathway between closed and open conformations of KcsA, the stability of the different channel states was assessed in molecular dynamics (MD) simulations. Repeated simulations (3 times 50 ns) of the structures, embedded in a lipid-bilayer membrane, were performed. The root-mean-square deviation (RMSD) of the backbone atoms without loops of all three channel states is less than 2 Å (Figure S1). The stability of the closed state is similar to previous values reported in literature [28], [29]. Moreover, the RMSD of the intermediate state is comparable to the two other states with a RMSD of 1.75 Å.

### Activation gating simulated by essential dynamics

To investigate the activation pathway, the backbone atoms of closed and open structures without loops were compared by principal component analysis (PCA). The resulting eigenvector (EV) was used to enforce the transition between the two states. Thus, the ED simulation is a free MD simulation, with all coordinates equilibrating except for one coordinate that is biased to drive the gating transition. Ten opening and ten closing ED simulations, all of them lasting for 20 ns, were carried out. In the following paragraphs, results of opening simulations are explained in detail. Since similar observations were also found in the reversed direction, results for the closing runs are summarized at the end of this section and corresponding figures are shown in the supplemental material.

The conformational changes during the ED opening simulations were analyzed by monitoring the RMSD as a function of time (Figure 1A). The deviation from the target structure (open conformation, pdb identifier: 3f7v) was measured over time. The difference between the starting and target structure is 4 Å. In all ten opening ED simulations, the RMSD values steadily decreased and reached final values between 1.35 and 2.20 Å, indicating that all simulations reached the open state. Successful opening is defined by a decrease of the RMSD to approximately 2 Å compared to the target structure. For simplicity, the average RMSD and standard deviation of the ten simulations were calculated. On average, a final RMSD of 2 Å as shown in Figure 1A was reached. The standard deviation indicates that in the first 11 ns, the RMSD values of the simulations did not vary. However, in the subsequent simulation time at which the simulations reached the target structure, the RMSD of the ten simulations showed wider distribution.

To investigate the conformational states of the end structures, the deviation of the Cα atoms from the target structure was analyzed. An average structure of the ten ED simulations was generated which exhibits minimal RMSD (Figure 1B). This average structure revealed that ED simulations were able to reach the target structure. Figure 1B shows the color coded deviation of each Cα atom from the open structure. As expected, the TM1 and P-helices displayed a very modest RMSD deviation of 0.05 Å to the target structure since there are no conformational changes in these regions during activation gating. In contrast, deviations up to 3 Å were found in the C-termini of the TM2 helices, which undergo large conformational changes during channel opening. Additionally, large deviations were found in the loop regions due to the high mobility of loops. Investigations on the loop region (amino acid G56) showed that mutations did not influence gating [30], [31]. Thus, the loops were not investigated further.

**Figure 2:**
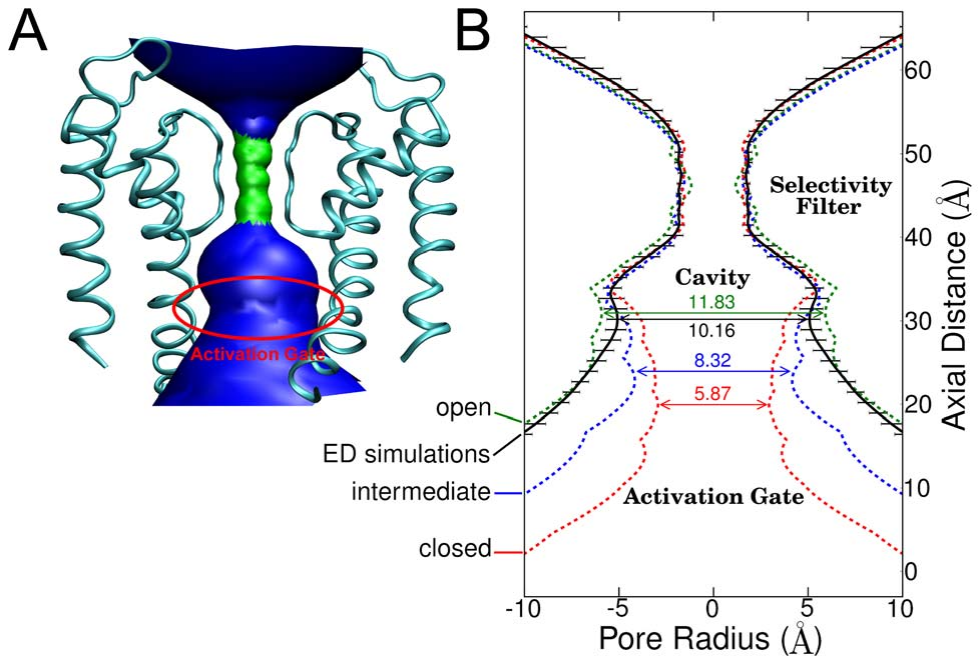
Pore radius profiles derived from backbone atoms of channel states. A) 3D representation of the pore domain depicting the HOLE profile. For the sake of clarity, only two opposing SUs are shown. B) Comparison of the profiles formed by the closed (red dashed line), intermediate (blue dashed line), and open (green dashed line) crystal structures with the average of the ten ED simulation structures (black dashed line). The subtle differences of the ED simulation structures in the activation gate region are indicated as standard deviation by error bars.

The program HOLE [32] was used to calculate the activation gate radius profiles (Figure 2) of the backbone atoms of different channel states. In the closed conformation, the constriction of the activation gate features a diameter of 5.9 Å. In the intermediate state, the diameter of the constriction site is 8.3 Å. In the open conformation, the activation gate diameter expands to 11.8 Å. The diameter of the activation gate in the ED simulations reached 10.7 Å on average. The shape of the pore radius profile of the end structures obtained from ED simulations matched the essential features of the profile of the open crystal conformation, further indicating that the simulation derived structures adopted the open state.

The major motions of opening were also observed in the reversed direction during closing (see Figure S2). However, only seven out of ten ED simulations successfully closed (RMSD<2.3 Å). Careful inspection revealed that the underlying reason for unsuccessful closure of three runs was partial unwinding of single TM2 helices. This observation may suggest that optimal packing of helices at the bundle crossing region is important for channel closure.

### Coupling between activation gate and SF

**Figure 3:**
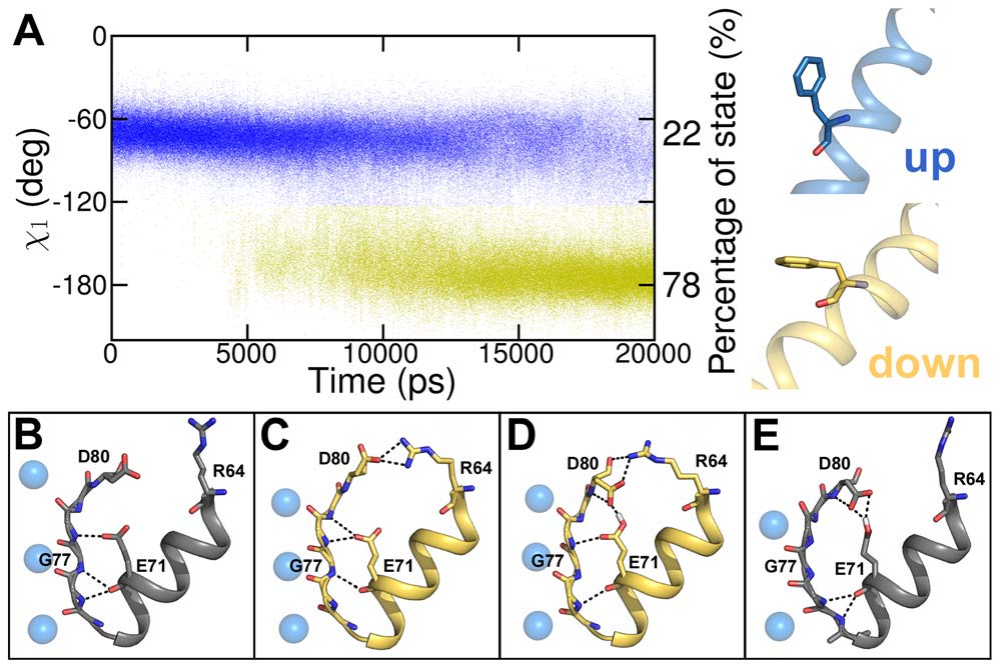
Conformational changes of F103 during activation gate opening and SF conformations of channel states. A) Analysis of χ_1_ angle dynamics of F103 (in ten opening ED simulations). Changes of the F103 orientation (χ_1_ angle) were measured over time. An angle of −70° indicates the “up” state (blue) while an angle of −180° represents the “down” state (yellow). The percentage of state was calculated from the end states at 20 ns of the ten ED simulations. B) SF and P-helix of the closed crystal structure (gray). Blue spheres represent K^+^ ions. C) SF and P-helix at the end of the 20 ns ED simulation structure with deprotonated E71 (yellow). D) SF and P-helix at the end of the 20 ns ED simulation structure with protonated E71 (yellow). E) SF and P-helix of the open inactivated crystal structure (gray). The G77 conformation defines the SF state as it was shown by Cuello et al. [12].

As described in the method section, no forces were applied to the side chains in the simulations. Hence, the simulations allowed investigations of the rotameric side chain changes coupled to gating. A phenylalanine, F103, present in the TM2 helices of KcsA, was shown to change its rotameric state upon activation gating [12], [21] and affecting the SF conformation [33], [34]. Therefore, the χ_1_ angle dynamics in the ten ED simulations were analyzed (Figure 3A). F103 can adopt two different rotameric states which are called “up” (χ_1_ angle of −55 to −72°) and “down” state (χ_1_ angle of −166 to −185°). In the first 5 ns of the opening ED simulations, F103 was stable in the up state. Subsequently, the conformational changes of the channel allowed F103 to adopt the down state. The F103 amino acids switched from the up to the down state over the next 15 ns. In most of the cases, this change was irreversible. Once F103 was in the down state, it was not able to switch to the up state again. After 20 ns, 78% of all F103 were in the down state. To validate if the F103 rotameric changes occurred because of activation gating, dihedral angles of unbiased open and closed state MD simulations were analyzed (data not shown). In the open state, all F103 of the three 50 ns MD simulations were in the down state. In the closed conformation, F103 showed more flexibility. Initially in the up state, the F103 was able to change to the down state; however, the up state is observed more frequently. This finding is in agreement with adiabatic energy maps of Pan et al [34] and a study by Cuello et al [33]. The dynamic behavior of F103 in the closing ED simulations is shown in Figure S2. In the first 2 ns, F103 was stable in the down state. Subsequently, F103 can adopt both up and down states as expected from the energy maps of Pan et al [34].

Despite different SF conformations in the closed and open crystal structures (actived vs. inactivated), the SF in all ten opening simulations did not adopt the inactivated conformation as seen in the crystal structure (pdb identifier: 3f7v; Figure 3B–E). The stability of the SF of the ED derived open conformation is further supported by a 100 ns free MD simulation, where no changes in the filter were observed. Previous studies reported that side chain hydrogen bonds between D80 and a protonated E71 promote inactivation of the SF [35]–[37]. Hence, we performed ED simulations with protonated E71 amino acids and analyzed the SF conformation. These simulations revealed similar conformations, irrespective of the protonation state. This conformation might be influenced by the ion occupancy in the filter. The ions were located at the most favored positions S0, S2, and S4 since the simulations started from a conductive state [38].

### Free energy profile of activation gating

**Figure 4:**
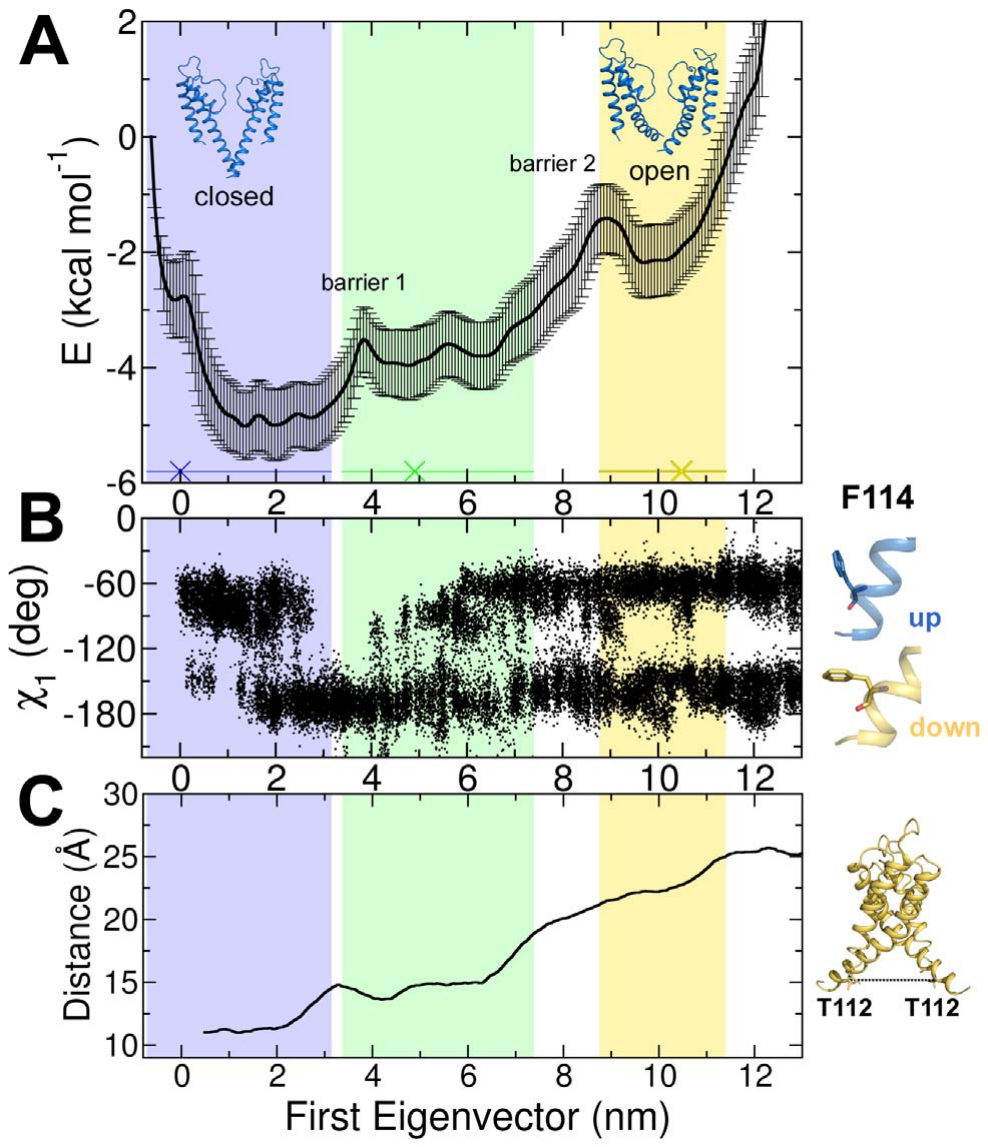
Free energy profile of gate opening. The blue, green, and yellow shades depict the sampling of the closed, intermediate, and open structures along the first EV. A) Free energy profile of the activation gate opening derived from 3.9 µs umbrella sampling simulations. The X marks indicate the positions of the crystal structures. B) χ_1_ angle dynamics of all four F114 during activation gating. C) Distance between opposite T112 as a measure of pore opening.

Umbrella sampling was employed to investigate the free energy landscape of activation gating (Figure 4A). The ED simulation with the lowest RMSD was used for a subsequent PCA calculation and thereof the first EV was employed as reaction coordinate. MD simulations of closed, intermediate, and open states were projected onto this reaction coordinate to determine sampling regions of the crystal structures. Three main energy wells, separated by two energy barriers, were identified. The first energy well, which is sampled by the closed state, is located at −0.7 to 3.1 nm. The intermediate state is sampled at the adjacent energy well, separated by a small energy barrier at 4 nm (barrier 1) from the closed state. Broad sampling of the intermediate conformation was observed, ranging from 3.4 to 7.4 nm. The subsequent large energy barrier at 9 nm (barrier 2) separates the open conformation from the intermediate state. The open conformation samples a relatively small energy well ranging from 8.8 to 11.4 nm. Next, we investigated the underlying structural rearrangements shaping the energy wells and barriers.

### Local structural rearrangements correspond to energy barrier 1

**Figure 5:**
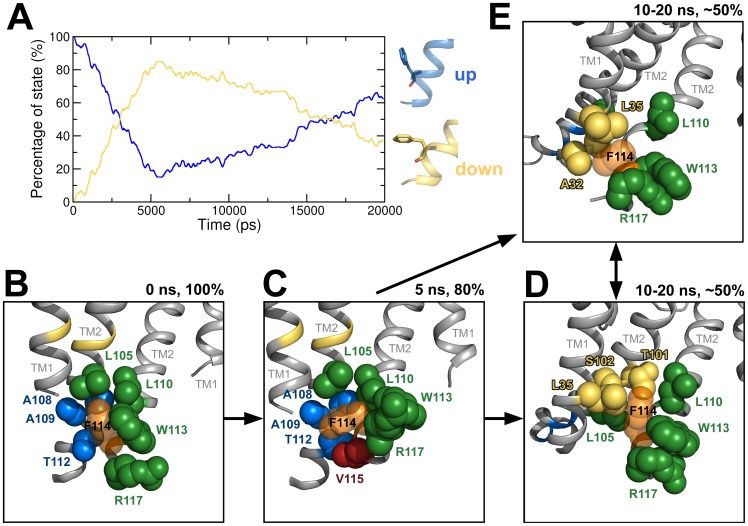
Analysis of χ_1_ angle dynamics of F114 and influence on packing. A) Percentage of F114 in the up (blue) and down (yellow) state over time. Packing of the F114 (transparent orange spheres) in the closed conformation (B), the transition state (C), the open states with F114 in the up (D) and the down state (E). Amino acids interacting in all states are shown in green. Interacting amino acids in the closed/open/transition state are represented in blue/yellow/red.

By analyzing the dihedral angles of all side chains, a single residue in the helix bundle crossing region was identified (F114) whose conformational changes correspond to the first energy barrier (Figure 4B). This unique rotameric pattern of F114 was observed in all ten opening ED simulation runs suggesting that this pattern was essential for activation gating (Figure 5A). In the early stage of activation gating (after 5 ns), 80% of all F114 changed from an up state (χ_1_ angle of −55 to −72°) to a down state (χ_1_ angle of −166 to −185°). After the change to the down state, a rigid phase from 5 to 10 ns was observed. Subsequently, F114 regained its flexibility. This suggests that the first flip of F114 and the changes in interacting amino acids may cause energy barrier 1. Consequently, interacting amino acids were analyzed in more detail. Figure 5B–E depicts residues that interact with F114 over time. Residues L110, W113, and R117 of TM2 and L105 of the adjacent TM2 helix interacting in all states are shown in green. Additional interacting amino acids in the closed state were A108, A109, and T112 of the adjacent TM2 (Figure 5B). In the rigid transition state (Figure 5C), additional interactions to V115 were observed. In the open state, interactions with T101 and S102 of the neighboring TM2 were found. When F114 occupied the down state, it was in close contact with A32 of the adjacent TM1 helix. F114 interacted with L35 (adjacent TM1 helix) independently of the rotameric state, indicating a specific interaction pattern. The importance of the F114 and adjacent amino acids is supported by experimental mutation studies (see section “relation to experimental data”).

The dynamical behavior of the F114 side chain is further supported by free MD simulations of the open and closed state. In the open state, 75% of the 12 F114 side chains in the MD simulations adopted the down state. Flipping between the two states occurred as a rare event, indicating that the F114 side chains showed high stability over 50 ns. An increased flexibility of F114 was observed in the closed state. Although 80% of the F114 side chains adopted the initial up state, flipping between the two states was observed frequently. Nevertheless, the specific rotameric pattern of F114 as seen during the ED simulations did not occur, indicating that this rotameric pattern is unique for activation gating. Additionally, these analyses showed that not only F103 but also F114 is allowed to adopt two rotameric states in the closed conformation.

### Global conformational changes of TM2 correspond to energy barrier 2

Cα-Cα distances between two opposite T112 residues (TM2) as a measure of activation gate opening (as proposed by Cuello et al [12]) were found to correlate with the energy barriers (Figure 4C). This measurement allows direct comparison of ED derived conformational states (closed, intermediate, and open) to the crystal structures. At the first energy barrier, an initial conformational change of the activation gate from 12 Å to 14 Å was observed correlating to structural rearrangements of F114. In the subsequent plateau phase of opening, a good correlation with the energy wells of the intermediate structures was observed. The second energy barrier is linked to a distance increase of 8 Å between the two opposing T112 residues. This suggests that the second energy barrier is mainly caused by global conformational changes of TM2. To further test the significance of this two-phasic activation gate opening, the T112 distances of all ten opening ED simulations were analyzed. Again, a two-phasic gating with global conformational changes at 4 to 5 ns and at 7.5 to 16 ns was found (Figure S3). These findings are in line with previous computational studies, which showed that the main opening of the gate occurs after an initial unlock from the closed state by structural rearrangements of amino acids [18], [21]. Additionally, simulations in the reverse direction showed similar local and global structural rearrangements in inverse order supporting the validity of the simulations.

### Relation to experimental data

The transition pathways obtained by the ED simulations are in good agreement with experimental data. First, the simulations are able to sample the intermediate crystal structure (pdb identifier: 3f7v; green shaded energy well in Figure 4A) [12], which was not included in our ED simulation protocol. Secondly, as expected [13], KcsA crystal structures 1k4c, 3f7v, and 3fb6 occupy energy wells in our calculated energy profile (Figure 4A). Thirdly, the energy profile indicates that the pore is intrinsically more stable in the closed conformation. This observation is supported by experimental studies on potassium channels [39]–[41], although it should be noted that the latter two studies were carried out on shaker-like channels, rendering the comparison indirect. Further, residues involved in pH sensing of KcsA were not included in the simulated system, which may also affect stability.

Simulations support the hypothesis that the F114 conformational changes are crucial to trigger initial activation gating. Mutational studies have shown the important role of the tightly packed helix bundle crossing region including F114. Several mutations in this region revealed a destabilization of the closed conformation [39], [42]. The fact that F114 is conserved in many K^+^ channels additionally underlines the importance of this aromatic amino acid for channel function [40], [43]–[46]. Mutational analysis of interacting amino acids in the open state like L35, T101, and T102 (analyzed in Shaker [40], [47]) or A32 would be of great interest and may lead to new insights into the packing of F114 in the open state.

### Lipid interactions of TM2 helices during activation gating

**Figure 6:**
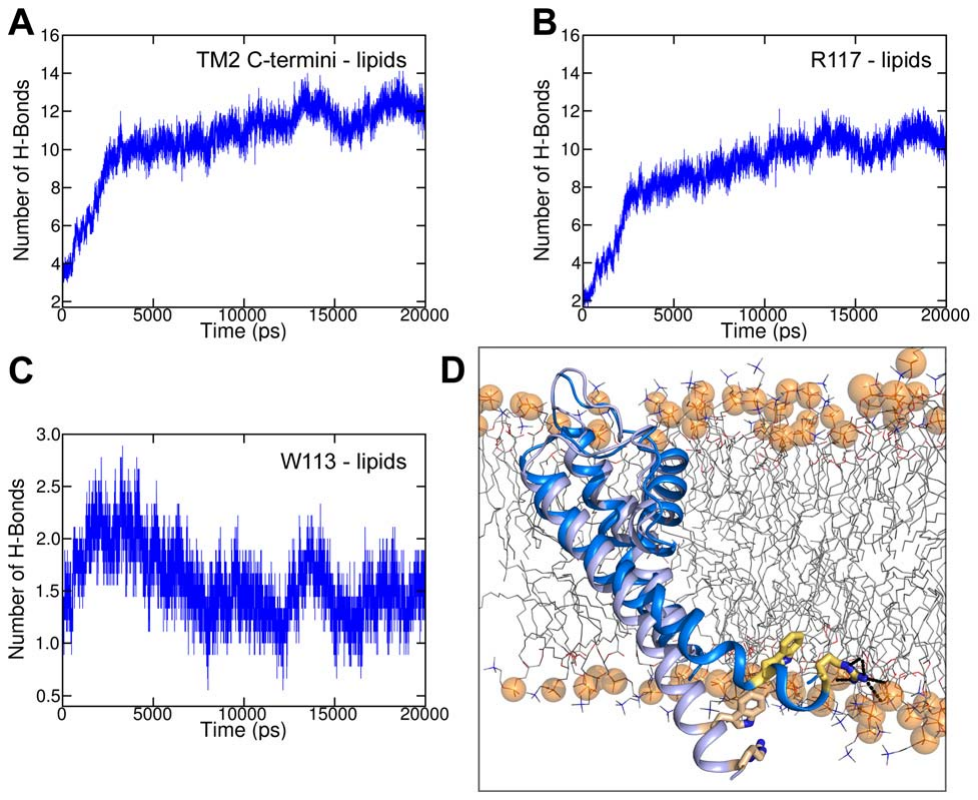
Lipid interactions of TM2 helices during activation gate opening. A) Average number of H-bonds between H-bond forming residues (W113 and R117) of the C-terminal TM2 helices and lipid head groups was measured over time. B) Average number of H-bonds of R117 with lipids. C) Average number of H-bonds of W113 with lipids. D) Representation of one SU in the closed (light blue) and open (marine blue) conformation with lipids. H-bond forming residues W113 and R117 are shown as yellow sticks. Lipids are depicted as gray lines while phosphate groups are shown as orange spheres. Dashed black lines represent H-bonds.

Since the C-terminus of the TM2 helices moves from a water environment towards the lipid/water interface during activation gating, interactions between the TM2 helices and lipids were investigated. Analyses revealed that the number of hydrogen bonds between the hydrogen bond forming residues W113 and R117 and the lipid head groups increased during gate opening (Figure 6). This indicates that the C-terminus of TM2 moved towards the inner leaflet of the bilayer membrane while hydrogen bonds are mainly formed between R117 and the phosphate groups of the lipids. A decrease of hydrogen bonds was found for the closing simulations (Figure S4) while TM2 moves back from the lipid environment to the water environment.

### Cooperativity of activation gating

**Figure 7:**
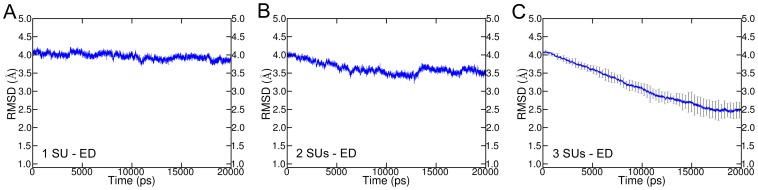
RMSD of cooperativity simulations. ED was applied on one (A), two (B), and three (C) SUs. The open structure was used as reference. For simulations with ED applied on one SU and two SUs, only one simulation each was performed. For ED simulations on three SUs, the average of the backbone RMSD without loops of ten simulations was measured. Standard deviation is indicated by the error bars.

ED simulations were applied on one, two, and three SUs, respectively, while the other SUs were allowed to move freely. Simulations revealed that at least three SUs are necessary to open the activation gate. RMSD analyses of simulations with the ED method applied on one and two SUs showed that there was only a slight decrease in RMSD over time suggesting that the channel remained in the closed state. However, simulations with the ED method applied on three SUs revealed that the end structures deviated 2.5 Å from the target structure (Figure 7). Cooperativity analyses of ED simulations presented in this study support previous studies on cooperativity of potassium channels in general [48]–[51] and of the pore domain in particular [19], [21], [52], [53]. Our simulations indicate that movement of one SU or two SUs is insufficient to open the gate. However, opening of three SUs is sufficient to obtain an open gate structure. Comprehensive investigations on cooperativity are subject of further studies.

### Conclusion

The results presented here show that the ED simulation approach successfully sampled transition pathways between closed and open states of an ion channel on the nanosecond time scale and allowed investigations on activation gating. There is good agreement between our investigations and previous experimental and computational studies, supporting the validity of this approach. The simulations provided new insights into conformational changes during gating and revealed that activation gating occurs as a two phase process. Additionally, investigation of the energy landscape allowed the correlation of conformational changes to energy barriers at the atomistic level. The first phase, in which local structural rearrangements in the helix bundle crossing region take place, correlates to a small energy barrier. The second phase was found to correlate with a large second energy barrier. During this phase, the main conformational changes of the TM2 helices, which occur upon gating, were observed.

In addition, we showed the feasibility of the ED approach to study the cooperativity of activation gating. The simulations suggest that individual SUs cannot open the activation gate. Rather, several SUs have to move in a cooperative manner in order to open the gate.

We expect that ED simulations will be useful for further investigations including the analysis of gating sensitive mutations. This is of special interest with regard to inherited channelopathies. Furthermore, we expect that these simulations will be valuable for studies on drug binding with different channel states.

## Methods

### Simulation setup

The closed (pdb identifier: 1k4c) [54] and open (pdb identifier: 3f7v) [12] crystal structures were used as starting conformations for the ED simulations. Additionally, they were subject to free MD simulations to assess the stability and the side chain dynamics. Free MD simulations of the intermediate conformation (pdb identifier: 3fb6) [12] were performed to investigate the sampling region of the structure along the transition pathway. Since the helices of the open conformation were not crystallized to the same extent as in the closed state (seven amino acids are missing at the beginning of TM1 and six amino acids at the end of TM2), the helix-lengths of the closed crystal structure were adapted by deleting these amino acids. The Q117 in the crystal structure of the open conformation was mutated to arginine in order to obtain the wild type structure using Swiss-PdbViewer [55]. For the intermediate state, one helical turn on the C-terminus was added in PdbViewer to obtain the same length of the helices as for the closed and open conformation. The protein was embedded in an equilibrated membrane consisting of 280 dioleolylphosphatidylcholine (DOPC) lipids using the g_membed tool [56], which is part of the gromacs package. K^+^ ions were placed in the SF, as described previously [57], at K^+^ sites S0, S2, and S4, with waters placed at S1 and S3 of the SF [38]. Cl^−^ ions were added randomly within the solvent to neutralize the system. All simulations were carried out using the gromacs simulation software v.4.5.4 [58]. The amber99sb force field [59] and the TIP3P model [60] were employed for the protein and water, respectively. Lipid parameter for the DOPC membrane were taken from Siu et al [61]. During all simulations, the area per lipid was at 0.72 nm^2^ which is in good agreement with experimental values [62]. Electrostatic interactions were calculated at every step with the particle-mesh Ewald method [63] with a short-range electrostatic interaction cut off of 1.4 nm. Lennard-Jones interactions were calculated with a cut off of 1.4 nm. The LINCS algorithm [64] was used to constrain bonds, allowing for an integration step of 2 fs. The Nose-Hoover thermostat was used to keep simulation temperature constant by coupling (tau = 0.5 ps for equilibration simulations and tau = 0.2 ps during unrestrained simulations) the protein, lipids and solvent (water and ions) separately to a temperature bath of 310 K. Likewise, the pressure was kept constant at 1 bar by using the Parrinello-Rahman barostat algorithm with a coupling constant of 1 ps. Prior to simulation, 1000 conjugate gradient energy-minimization steps were performed, followed by 5 ns of equilibrium simulation in which the protein atoms were restrained by a force constant of 1000 kJ mol^−1^ nm^−2^ to their initial position. Lipids, ions, and water were allowed to move freely during equilibration.

### Molecular dynamics simulations

In order to assess the stability of the open, intermediate, and closed conformation of the KcsA channel, three 50 ns unrestrained MD simulations were carried out for each structure.

### Principal component analysis

The basic method of the PCA is described in detail elsewhere [65]. A trajectory consisting of the closed and the open conformation was built and used for PCA. Subsequently, the covariance matrix of the positional fluctuations of the TM1, P-helix, and TM2 backbone atoms was built up and diagonalized (loops were excluded from analysis). For the PCA, all four SUs (one, two, and three SUs for cooperativity investigations) of the homotetrameric channel were taken into account. Only one EV with a non-zero eigenvalue results from this PCA, which represents the difference vector between the open and the closed crystal conformation. This vector was used as reaction coordinate for ED simulations.

### Essential dynamics simulations

The ED technique [23], [24] can be used to simulate the conformational pathway between two crystal structures [26]. During simulation, the distance along the first EV was increased in fixed increments to drive the system from the closed to the open state and vice versa. It is important to emphasize that the EVs were obtained by PCA of the backbone atoms only and therefore did not contain any information on the side chains. For simulations, the equilibrated closed and open systems, respectively, consisting of the channel, lipid-membrane, ions, and water, were used as start positions. Helical restraints were applied to the last four C-terminal amino acids of the TM2 helix of each SU in order to prevent unwinding. All parameters were set as described above. Simulations were performed on the 20 ns timescale. Fixed increment linear expansion for each simulation step (2 fs) was set to 1.28e^−6^ nm in order that the target structure was reached after two thirds of the simulation time. For cooperativity investigations, fixed increment linear expansion was set to 1.89e^−7^ nm, 6.27e^−7^ nm, 9.24e^−7^ nm per step (2 fs) and was applied to one SU, two SUs, and three SUs, respectively.

### Umbrella sampling

The windows for the umbrella sampling simulation were taken from the ED simulation with the lowest RMSD. The first EV, which was derived from a PCA of the ED simulation, was used as a reaction coordinate. As this EV is dominant (its eigenvalue is more than an order of magnitude larger than the second largest), we assume that the transition pathway is sufficiently accurately covered by this mode. Along this reaction coordinate, 39 windows with the corresponding structures from the first ED simulation were chosen for umbrella sampling and simulated for 100 ns (Figure S5). 33 windows were simulated with a force constant of 1 kJ mol^−1^ nm^−2^. For six windows, the force constant was set to 100 kJ mol^−1^ nm^−2^ in order to obtain sufficient sampling of the energy barriers. In total, umbrella sampling was performed for 3.9 µs. The first 50 ns of each window were discarded for equilibration. The potential of mean force and the statistical errors of the activation gating energy profile were estimated by making use of the g_wham tool of gromacs and the integrated bootstrap analysis method [66]. The number of bootstraps was set to 50.

## Supporting Information

Figure S1 **Stability of KcsA channel states.** Backbone RMSD (without loops) of three independent MD simulations of closed (A), intermediate (B), and open state (C) was measured as a function of time.
(TIF)

Figure S2 **Analysis of ED closing simulations.** A) Average of the backbone RMSD without loops of seven closing ED simulations. The closed crystal structure was used as reference. The standard deviation is indicated by error bars. B) Conformational changes of F103 during activation gate closing. Analysis of χ_1_ angle dynamics of F103 of the seven ED simulations was performed. Changes of the F103 orientation was measured as χ_1_ angle over time. An angle of −70° indicates the “up” state (blue) while an angle of −180° represents the “down” state (yellow). C) χ_1_ angle dynamics of F114 are shown as percentage of F114 in the up (blue) and down (yellow) states over time.
(TIF)

Figure S3 **Average of the Cα-Cα T112-distances of all ten ED simulations.** The standard deviation is indicated by error bars.
(TIF)

Figure S4 **Lipid interactions of TM2 helices during activation gate closing.** A) Average number of H-bonds between H-bond forming residues (W113 and R117) of the C-terminal TM2 helices and lipid head groups was measured over time. B) Average number of H-bonds of R117 with lipids. C) Average number of H-bonds of W113 with lipids.
(TIF)

Figure S5 **Histograms of the 39 umbrella sampling windows.** The six windows with peaks above 40000 were derived from umbrella sampling with a force constant of 100 kJ mol^−1^ nm^−2^ (default: 1 kJ mol^−1^ nm^−2^).
(TIF)
