# Supplementary figures and images for: Probing the Energy Landscape of Activation Gating of the Bacterial Potassium Channel KcsA
Source: PLoS Comput Biol. 2013 May 2;9(5):e1003058. doi: 10.1371/journal.pcbi.1003058 (PMC3642040; doi:10.1371/journal.pcbi.1003058)

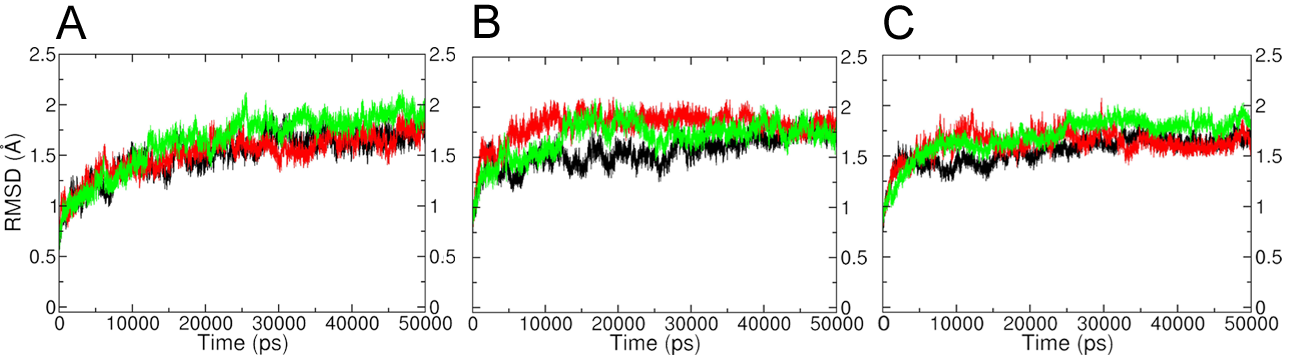

Supplement: Figure S1 — Stability of KcsA channel states. Backbone RMSD (without loops) of three independent MD simulations of closed (A), intermediate (B), and open state (C) was measured as a function of time. (TIF) [file pcbi.1003058.s001.tif]

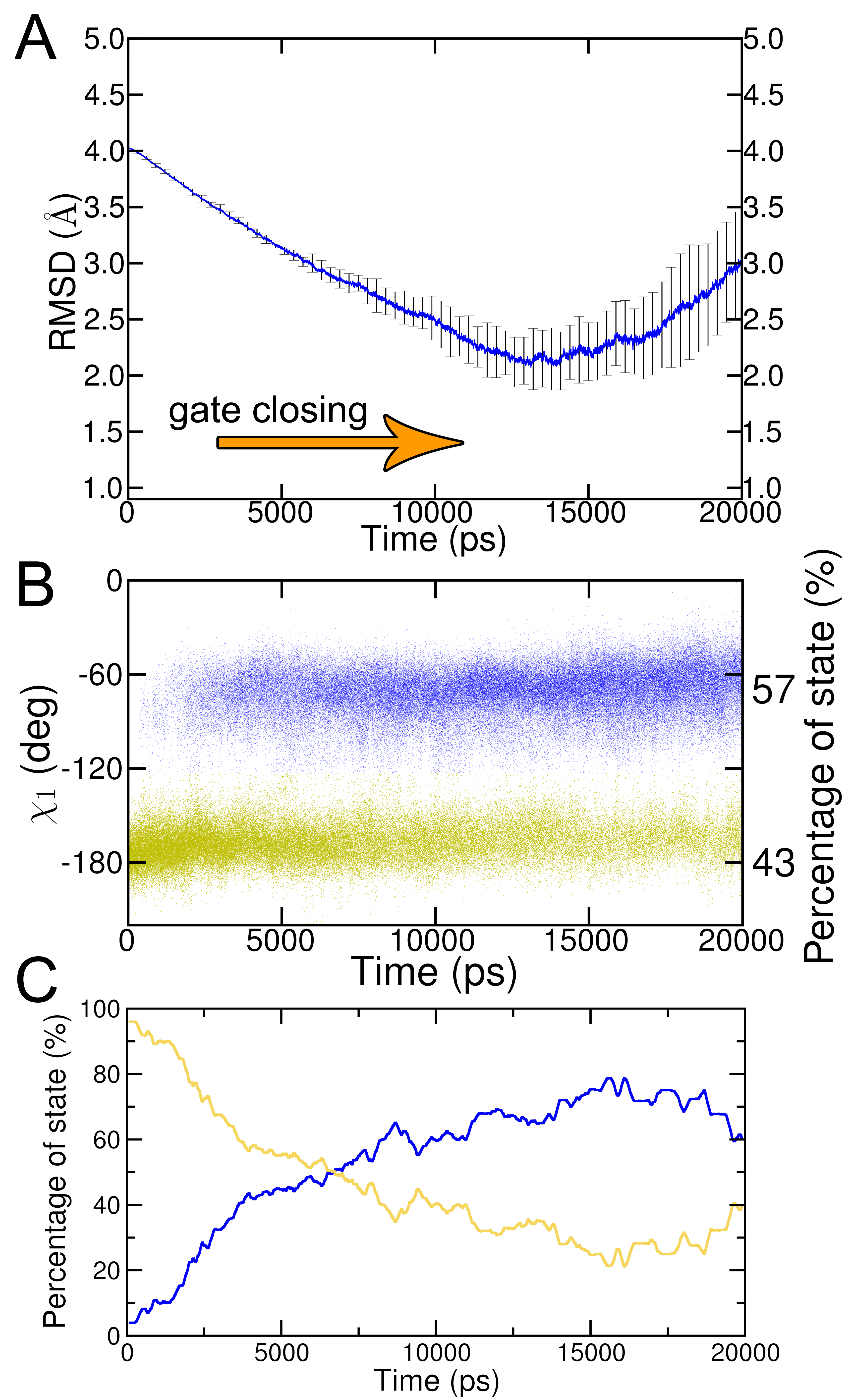

Supplement: Figure S2 — Analysis of ED closing simulations. A) Average of the backbone RMSD without loops of seven closing ED simulations. The closed crystal structure was used as reference. The standard deviation is indicated by error bars. B) Conformational changes of F103 during activation gate closing. Analysis of χ1 angle dynamics of F103 of the seven ED simulations was performed. Changes of the F103 orientation was measured as χ1 angle over time. An angle of −70° indicates the “up” state (blue) while an angle of −180° represents the “down” state (yellow). C) χ1 angle dynamics of F114 are shown as percentage of F114 in the up (blue) and down (yellow) states over time. (TIF) [file pcbi.1003058.s002.tif]

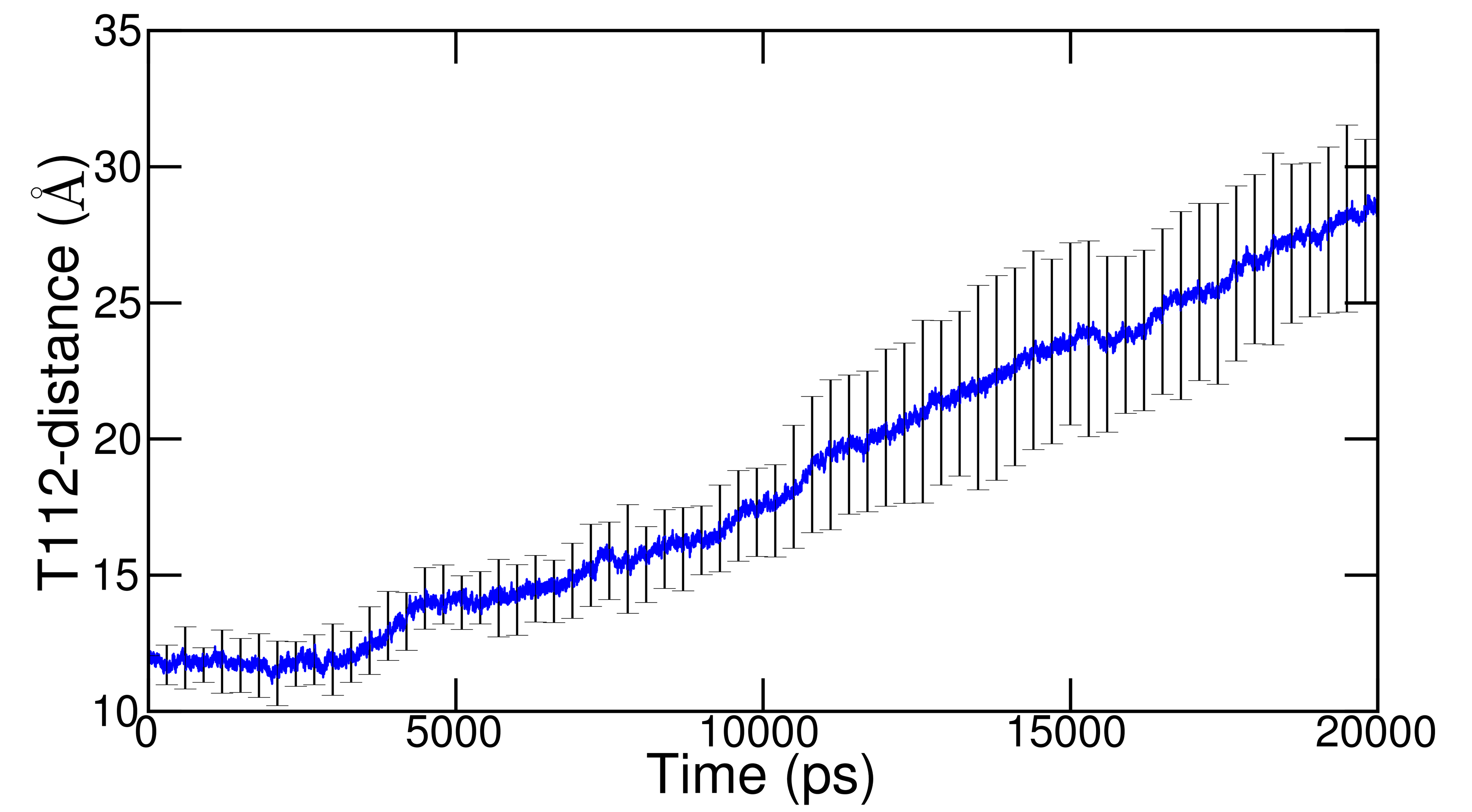

Supplement: Figure S3 — Average of the Cα-Cα T112-distances of all ten ED simulations. The standard deviation is indicated by error bars. (TIF) [file pcbi.1003058.s003.tif]

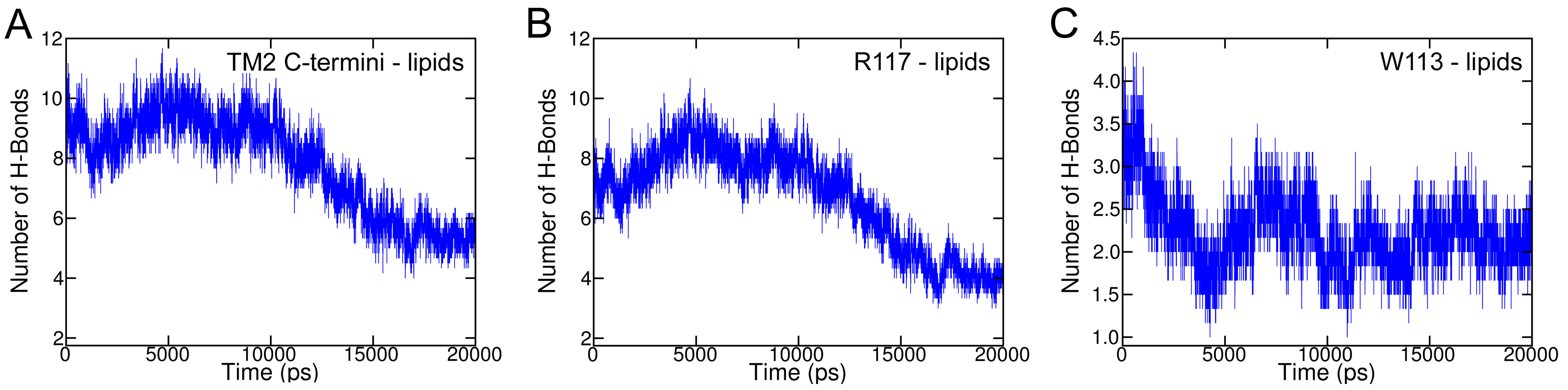

Supplement: Figure S4 — Lipid interactions of TM2 helices during activation gate closing. A) Average number of H-bonds between H-bond forming residues (W113 and R117) of the C-terminal TM2 helices and lipid head groups was measured over time. B) Average number of H-bonds of R117 with lipids. C) Average number of H-bonds of W113 with lipids. (TIF) [file pcbi.1003058.s004.tif]

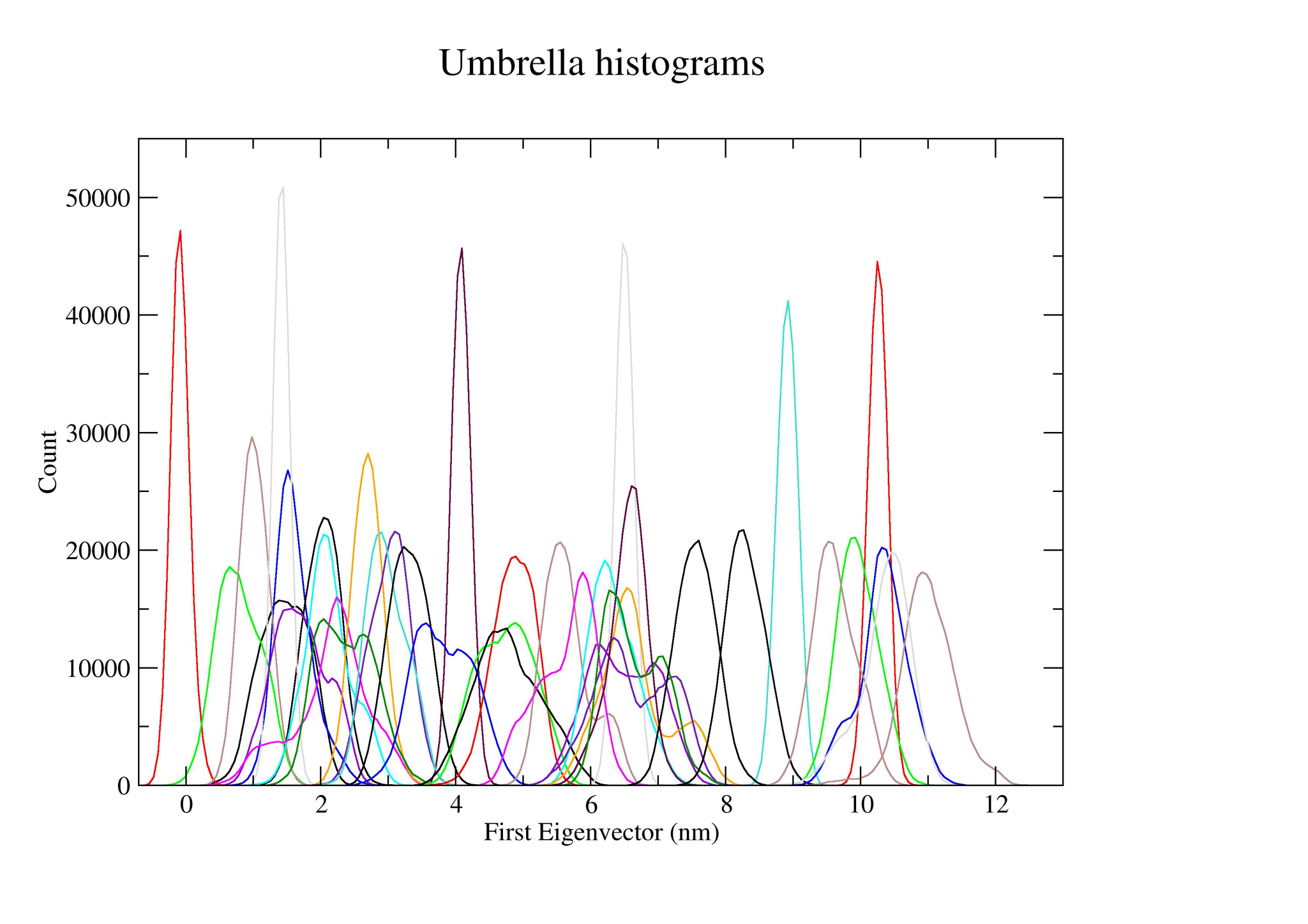

Supplement: Figure S5 — Histograms of the 39 umbrella sampling windows. The six windows with peaks above 40000 were derived from umbrella sampling with a force constant of 100 kJ mol−1 nm−2 (default: 1 kJ mol−1 nm−2). (TIF) [file pcbi.1003058.s005.tif]
